# Affinity Improvement of a Therapeutic Antibody by Structure-Based Computational Design: Generation of Electrostatic Interactions in the Transition State Stabilizes the Antibody-Antigen Complex

**DOI:** 10.1371/journal.pone.0087099

**Published:** 2014-01-27

**Authors:** Masato Kiyoshi, Jose M. M. Caaveiro, Eri Miura, Satoru Nagatoishi, Makoto Nakakido, Shinji Soga, Hiroki Shirai, Shigeki Kawabata, Kouhei Tsumoto

**Affiliations:** 1 Medical Genome Sciences, Graduate School of Frontier Sciences, The University of Tokyo, Kashiwa, Chiba, Japan; 2 Department of Bioengineering, Graduate School of Engineering, The University of Tokyo, Bunkyo-ku, Tokyo, Japan; 3 Laboratory of Medical Proteomics, Institute of Medical Science, The University of Tokyo, Minato-ku, Tokyo, Japan; 4 Molecular Medicine Research Laboratories, Drug Discovery Research, Astellas Pharma Inc., Tsukuba, Ibaraki, Japan; 5 Department of Chemistry and Biotechnology, Graduate School of Engineering, The University of Tokyo, Bunkyo-ku, Tokyo, Japan; Monash University, Australia

## Abstract

The optimization of antibodies is a desirable goal towards the development of better therapeutic strategies. The antibody 11K2 was previously developed as a therapeutic tool for inflammatory diseases, and displays very high affinity (4.6 pM) for its antigen the chemokine MCP-1 (monocyte chemo-attractant protein-1). We have employed a virtual library of mutations of 11K2 to identify antibody variants of potentially higher affinity, and to establish benchmarks in the engineering of a mature therapeutic antibody. The most promising candidates identified in the virtual screening were examined by surface plasmon resonance to validate the computational predictions, and to characterize their binding affinity and key thermodynamic properties in detail. Only mutations in the light-chain of the antibody are effective at enhancing its affinity for the antigen *in vitro*, suggesting that the interaction surface of the heavy-chain (dominated by the hot-spot residue Phe101) is not amenable to optimization. The single-mutation with the highest affinity is L-N31R (4.6-fold higher affinity than wild-type antibody). Importantly, all the single-mutations showing increase affinity incorporate a charged residue (Arg, Asp, or Glu). The characterization of the relevant thermodynamic parameters clarifies the energetic mechanism. Essentially, the formation of new electrostatic interactions early in the binding reaction coordinate (transition state or earlier) benefits the durability of the antibody-antigen complex. The combination of *in silico* calculations and thermodynamic analysis is an effective strategy to improve the affinity of a matured therapeutic antibody.

## Introduction

The exquisite specificity and high affinity of antibodies are increasingly exploited for therapeutic and biotechnological purposes, such as in cancer immunotherapy, in diagnosis, and in molecular bio-sensors [1]–[3]. Biobetter, defined as the biomedicine successfully developed through functional and/or physicochemical improvement of a natural molecule, provides a promising strategy for the next generation of therapeutics [4]. However, the optimization of the physicochemical properties of an antibody without perturbing the affinity and specificity for the cognate molecule is a challenging endeavor. Progress in this field is accelerating, and successful examples of improved antibodies using biobetter strategies have been reported, such as higher affinity for the cognate, increased stability in solution, enhanced pharmacokinetics, diminished immunogenicity, or conjugation to drug delivery systems [5]–[9]. Nevertheless, to advance further the deployment of biobetter strategies in the design and preparation of improved therapeutics it is necessary to strengthen our understanding of the physicochemical properties of engineered antibodies [10].

The interaction surface of antibody-antigen complexes involves a large number of residues and water molecules establishing multiple non-covalent interactions that are difficult to quantify at the molecular and atomic levels. Although the evaluation of protein-protein binding energy remains a challenging task, progress in computing performance and force-field parametrization are rapidly advancing our predictive capabilities [11], [12]. Thus structure-based computational techniques are increasingly employed in the design of biotherapeutic antibodies [13].

**Figure 1:**
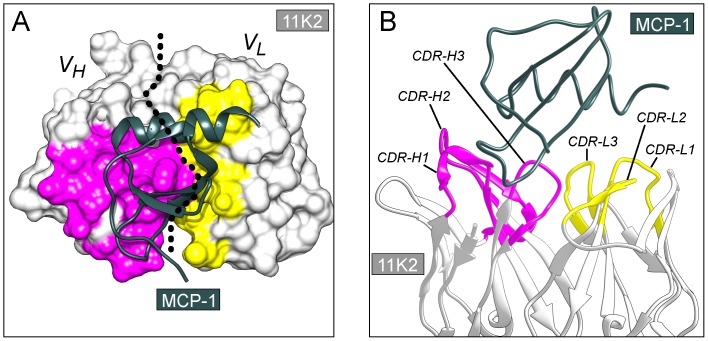
Crystal structure of 11K2 Fab in complex with its antigen MCP-1. The coordinates were retrieved from the PDB (accession code 2BDN). (**A**) Interaction surface of 11K2. The surface in magenta (V_H_) and yellow (V_L_) correspond to residues of the antibody interacting with the antigen. Other residues are shown in light gray. The dotted line indicates the boundary between V_H_ and V_L_ chains. The antigen is depicted in dark green. (**B**) CDR of the antibody. All the residues belonging to the CDR were subjected to virtual systematic mutagenesis. The ribbons in magenta and yellow belong to V_H_ and V_L_ chains, respectively. The dark green ribbons depict the antigen.

Herein, we report the improvement of the binding affinity of the mature antibody 11K2 for its target antigen MCP-1 (monocyte chemotactic protein-1), an important therapeutic target in inflammatory diseases such as arteriosclerosis [14], allergy [15], and rheumatoid arthritis [16]. The design of an optimized version of the antibody 11K2 with enhanced binding capabilities may improve its therapeutic value in the treatment of inflammatory diseases. Although the binding affinity of 11K2 for its antigen is very high (4.6 pM) [17], the analysis of the crystal structure of the antibody-antigen complex (PDB entry code 2BDN) suggests how to further increase the affinity by optimizing the complementary determining region (CDR) of the variable domain of the light chain (V_L_) (Figure 1) [18]. Indeed, the interaction surface between the CDR of the V_L_ of 11K2 and the antigen is 257 Å^2^, a value significantly smaller than that of the CDR of the variable domain of the heavy chain (V_H_) (511 Å^2^) [19]. Moreover, the evaluation of the thermodynamic properties of the engineered antibodies is a desirable goal towards increasing our predictive capabilities in future optimization strategies.

In this report we have calculated the binding energy between MCP-1 and a virtual library of systematic mutations at each of the 62 residues of the CDR of the antibody 11K2 by computational methods. The *in silico* calculations suggest that single-mutations carrying charged residues at specific locations favor the binding energy with the antigen. The affinity of the most promising candidates was determined experimentally by the technique of surface plasmon resonance (SPR), which identified several muteins with enhanced affinity for the antigen. The thermodynamic analysis reveals the fundamental mechanism explaining the superior binding capabilities of the optimized antibody. Our study highlights the benefits of combining *in silico* and *in vitro* methodologies for a more effective molecular design by biobetter strategies.

## Materials and Methods

### Comprehensive mutagenesis *in silico*

Each of the 62 residues belonging to the CDR of 11K2 (as defined by Kabat [20] and Chothia [21]) were subjected to systematic mutagenesis *in silico* with each of the other 19 natural amino acids (62×19  =  1,178 mutations). The initial coordinates of 11K2 in complex with MCP-1 were retrieved from the PDB (entry code 2BDN). For each mutation in the antibody, 100 randomized models were generated using the default parameters of the program MODELLER of the Discovery Studio Suite (Accerlys, San Diego, CA) [22]. Each model of the mutated antibody-antigen complex was optimized by a combination of simulated annealing followed by molecular mechanics minimization until the mean square gradient decreased below 0.01 kcal mol^−1^ Å^−2^. The interaction energy between antigen and antibody in each of the 117,800 models was calculated using the default values in the module Einteract of the package software MOE (Chemical Computing Group, Canada) using the AMBER99 force field after hydrogen atoms were explicitly added and minimized. We used the default dielectric constant (80.0), mimicking the behavior of water. Structural models of muteins lacking non-covalent interactions with the antigen were not considered for further analysis. For each mutation tested, the electrostatic and van der Waals interaction energies were summed up and applied as a surrogate to evaluate the affinity of the mutant for the antigen, as described previously [23]. As a reference we built 1,000 model structures of native antibody by optimizing the x-ray structure using the program MODELLER as explained above. Mutations displaying histograms with more favorable energy of interaction than that of wild type protein were selected for *in vitro* examination (H-L27R, H-L27K, H-N28D, H-N28Q, H-D31E, L-Y30K, L-N31R, L-N31K, L-S53D, L-S53E, L-T56D, L-T56E) (see a representative example in Figure S1).

### Expression and purification of MCP-1

The DNA encoding MCP-1 was synthesized by GeneArt (Regensbyrg, Germany) and sequence-optimized for expression in *Escherichia coli* (Table S1). The MCP-1 gene was expressed in a vector pET26b (Novagen) displaying a hexa-histidine tag at the C-terminus. The DNA sequence was confirmed by the dideoxy chain-termination method.

*E. coli* strain Rosetta2 (DE3) cells (Novagen) transformed with the expression vector of MCP-1 were grown at 28°C in 2× YT broth. Protein expression was induced by addition of 0.5 mM isopropyl β-D-1-thiogalactopyranoside when the optical density at 600 nm reached a value of 0.6. Cells were allowed to grow for an additional 16 h at 28°C. The cells were harvested by centrifugation at 8,000× *g* for 8 min and the pellet thus obtained was resuspended in 40 ml of a solution containing 0.5 M NaCl and 50 mM Tris-HCl at pH 8.0 (buffer A). Cells were subsequently lysed by the sonication method with an ultrasonic cell-disruptor instrument (Tommy) for 15 min (Output 7, Duty 50). A compact pellet containing the soluble intracellular components was obtained by centrifugation at 40,000× *g* for 30 min. The soluble fraction was collected and applied onto a Ni-NTA column (Novagen) equilibrated with buffer A. Protein was eluted with stepwise increase of imidazole (10, 20, 30, 50, 100, 200, and 300 mM) in buffer A. The fractions containing MCP-1 were pooled, and subjected to size exclusion chromatography using a HiLoad 26/60 Superdex 75-pg column (GE Healthcare) equilibrated with 50 mM Tris, NaCl 500 mM, EDTA 1 mM at pH 7.4.

### Expression and purification of 11K2 scFv

The DNA encoding the single-chain variable fragment (scFv) of 11K2 was chemically synthesized by GeneArt and sequence-optimized for expression in *E. coli* (Table S1). The 11K2 scFv construct was expressed in vector pUTE [24] displaying a hexa-histidine tag at the C-terminus. The DNA sequence was confirmed by the dideoxy chain-termination method.

Cells of *E. coli* strain BL21 (DE3) (Novagen) were transformed with the expression vector of 11K2 scFv and grown at 28°C in LB broth. Protein expression, cell harvesting, and cell lysis were performed as described above for MCP-1. A compact pellet containing the insoluble intracellular components was obtained by centrifugation at 7,500×*g* for 30 min. SDS-PAGE analysis and western blotting were conducted using the insoluble fraction (Figure S2). The soluble fraction was discarded. The insoluble fraction was then solubilized with 6 M guanidine-HCl, 0.5 M NaCl and 50 mM Tris-HCl overnight at 4°C. After solubilization, 11K2 scFv was purified in a Ni-NTA column (Novagen) as described above for MCP-1, except that the equilibration and elution buffers were supplemented with 6 M guanidine-HCl (denaturing conditions).

The purified antibody was refolded by the stepwise dialysis method [25]. Briefly, 11K2 scFv was diluted to 7.5 µM with 6 M Guanidine-HCl in 0.2 M NaCl, 50 mM Tris-HCl, 1 mM EDTA (pH 8.0), followed by stepwise dialysis to remove the denaturant. In order to increase the refolding efficiency, 0.2 M L-Arg-HCl was added to the dialysis solution to minimize protein aggregation when the concentration of guanidine-HCl decreased to 1–0.5 M. The refolded antibody was further purified on a HiLoad 26/60 Superdex 75-pg column equilibrated with a solution containing 0.2 M NaCl, 50 mM Tris-HCl and 1 mM EDTA (pH 8.0). The same protocol was employed for the purification of the antibody muteins.

### Binding Assays by SPR

The interaction between MCP-1 and wild-type 11K2 scFv (or muteins) was analyzed by SPR in a Biacore T200 instrument (GE Healthcare). Research grade CM5 Biacore sensor chip (GE Healthcare) was activated by a short treatment with N-hydroxysuccinimide/N-ethyl-N’-(3-dimethylaminopropyl) carbodiimide hydrochloride, followed by immobilization of the antigen MCP-1 at a surface density of ∼220 RU. The activated groups on the surface of the sensor were subsequently blocked by injecting 100 µl of a solution containing 1 M ethanolamine. The kinetic data of the binding of 11K2 scFv to the antigen were obtained by injecting increasing concentration of antibody into the sensor chip at a flow rate of 30 µl/min. The measurements were carried out in PBS containing 0.005% (v/v) Tween-20. Contact time and dissociation time were 5 min and 20 min, respectively. Data analysis was performed with the BIAevaluation software (GE Healthcare). Association (*k_on_*) and dissociation (*k_off_*) rate constants were calculated by a global fitting analysis assuming a Langmuir binding model and a stoichiometry of (1∶1). The dissociation constant (*K*_D_) was determined from the ratio of the rate constants [26]:
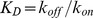



### Calculation of thermodynamic parameters

Changes in enthalpy (Δ*H°*) and entropy (Δ*S°*) were calculated from the slope and intercept, respectively, of the temperature dependence of the dissociation constant using the van’t Hoff approximation [27]:
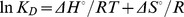
where *R* is the gas constant and *T* is the absolute temperature.

The activation energy parameters were obtained from the temperature dependence of the association rate constant following the Eyring approximation:
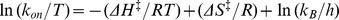



where *k_on_* is the association rate constant, Δ*H* ^‡^ is the activation enthalpy, *R* is the gas constant, *T* is the absolute temperature, *ΔS^‡^* is the activation entropy, *k_B_* is the Boltzmann’s constant, and *h* is the Plank’s constant.

## Results

### Computational Selection of Favorable Mutations

**Figure 2:**
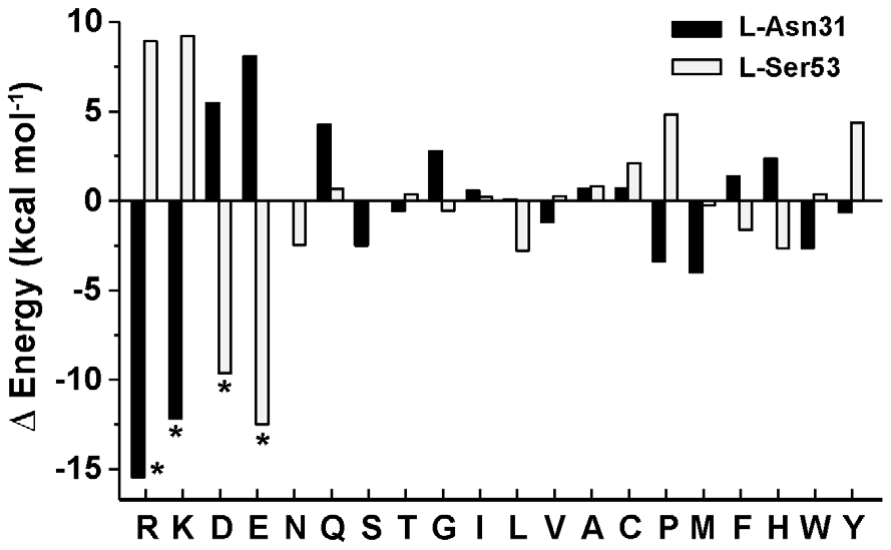
Mutagenesis *in silico*. Average energy values (as the sum of electrostatic and van der Waals energies) of all possible mutations of two different residues (L-Asn31 and L-Ser53) of antibody 11K2 with respect to wild-type. Negative values suggest higher affinity between the mutated protein and the antigen. The mutations indicated by the asterisks were selected for further examination by SPR.

To improve the high-affinity of antibody 11K2 for its antigen MCP-1 we selected potentially favorable mutations from a virtual screening (*in silico*) consisting of 1,178 single mutations (19 mutations for each of the 62 residues composing the CDR loops). The force field AMBER99 as implemented in the software MOE was used to perform 100 energy minimizations of each mutation (total was 117,800 minimizations), and the corresponding values of energy were subsequently plotted as histograms (Figure S1). The overall shape of the histogram and the median were used to estimate the relative efficacy of each mutation with respect to wild-type antibody. The relative energies of two representative sets of virtual mutations are shown in Figure 2. The residues examined in the example are L-Asp31 and L-Ser53, which are located in the first and second CDR of the V_L_ chain, respectively. In a large number of virtual mutations, the change of energy is comparatively small (less than ±3 kcal mol^−1^). The greatest differences are observed in virtual muteins displaying charged residues (Arg, Lys, Glu or Asp). In some cases, the substitution by a charged residue increases the attractive energy, whereas in other cases the value of energy becomes clearly unfavorable. For example, the calculated energy of mutein L-N31R is clearly more advantageous for binding the antigen than that of wild-type antibody (Δ*E* ^L−N31R^  =  −15.4 kcal mol^−1^), whereas the relative change of energy in mutein L-S53R is clearly destabilizing (Δ*E* ^L−S53R^  =  8.9 kcal mol^−1^). Mutations displaying very favorable changes of energy in Figure 2 were selected for experimental validation by the technique of SPR (muteins L-D31R, L-D31K, L-S53D, and L-S53E). The following muteins were also selected from the whole virtual screening and examined by SPR: H-L27R, H-L27K, H-N28D, H-N28Q, H-D31E, L-Y30K, L-T56D, L-T56E. We note that 11 muteins from a total of 12 selected mutations involved charged residues (92%).

### Evaluation of the affinity by SPR

**Figure 3:**
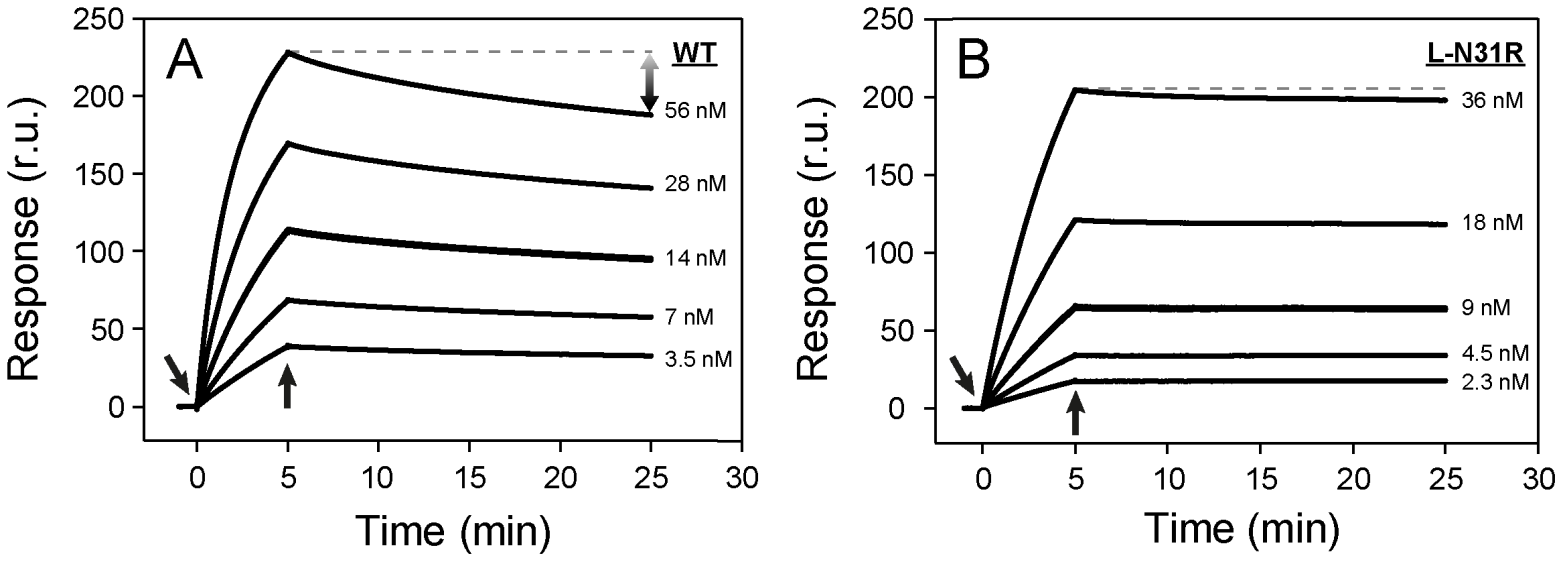
Binding sensorgrams. (**A**) Binding of wild-type 11K2 to its antigen MCP-1. (**B**) Binding of mutein L-N31R to MCP-1. The arrows pointing downward indicate injection of running buffer with 11K2 antibody. The arrows pointing upward correspond to the injection of buffer with no antibody. The response signal is proportional to the amount of scFv 11K2 binding to a chip decorated with antigen MCP-1. The straight dotted line at the top curve in each panel is drawn to appreciate the slower dissociation rate of the mutein with respect to the wild-type protein. The concentration of antibody is given in each panel.

The kinetic rate constants of the binding of wild-type scFv 11K2 (and single-muteins) to immobilized antigen MCP-1 were examined by SPR (Figure 3, Table 1). The injection of the antibody to a surface decorated with antigen produces an increase of the SPR signal that is correlated with the association constant rate (*k_on_*) (Figure 3A). The dissociation rate constant (*k_off_*) is determined from the signal decay after depleting the solutions from antibody. The values of *k*_on_ and *k*_off_ determined at 25°C were 14×10^4^ M^−1^ s^−1^ and 1.0×10^−4^ s^−1^, respectively, corresponding to a dissociation constant (*K*_D_) of 0.8 nM. The small values of *k_off_* indicate slow dissociation rates − a clear evidence of tight binding of the antibody to the antigen.

**Table 1:** Kinetic parameters of binding of scFv 11K2 to MCP-1 at 25°C.

Protein	*k_on_* (M^−1^ s^−1^)	*k_off_* (s^−1^)	*K_D_* (nM)	*K_D_* ^WT^/*K_D_* ^mut^	Improved
**WT**	14×10^4^	1.0×10^−4^	0.80	1	-
**H-L27R**	14×10^4^	3.3×10^−4^	2.4	0.3	NO
**H-L27K**	11×10^4^	3.9×10^−4^	3.5	0.2	NO
**H-N28D**	1.6×10^4^	1.6×10^−4^	1.0	0.8	NO
**H-N28Q**	22×10^4^	19×10^−4^	8.5	0.09	NO
**H-D31E**	3.0×10^4^	1.5×10^−4^	5.0	0.16	NO
**L-Y30K**	13×10^4^	3.1×10^−4^	2.5	0.3	NO
**L-N31R**	13×10^4^	0.22×10^−4^	0.17	4.6	YES
**L-N31K**	34×10^4^	980×10^−4^	290	0.003	NO
**L-S53D**	9.0×10^4^	0.55×10^−4^	0.61	1.3	YES
**L-S53E**	7.3×10^4^	0.14×10^−4^	0.19	4.2	YES
**L-T56D**	22×10^4^	0.86×10^−4^	0.39	2.1	YES
**L-T56E**	10×10^4^	0.26×10^−4^	0.25	3.2	YES

**Figure 4:**
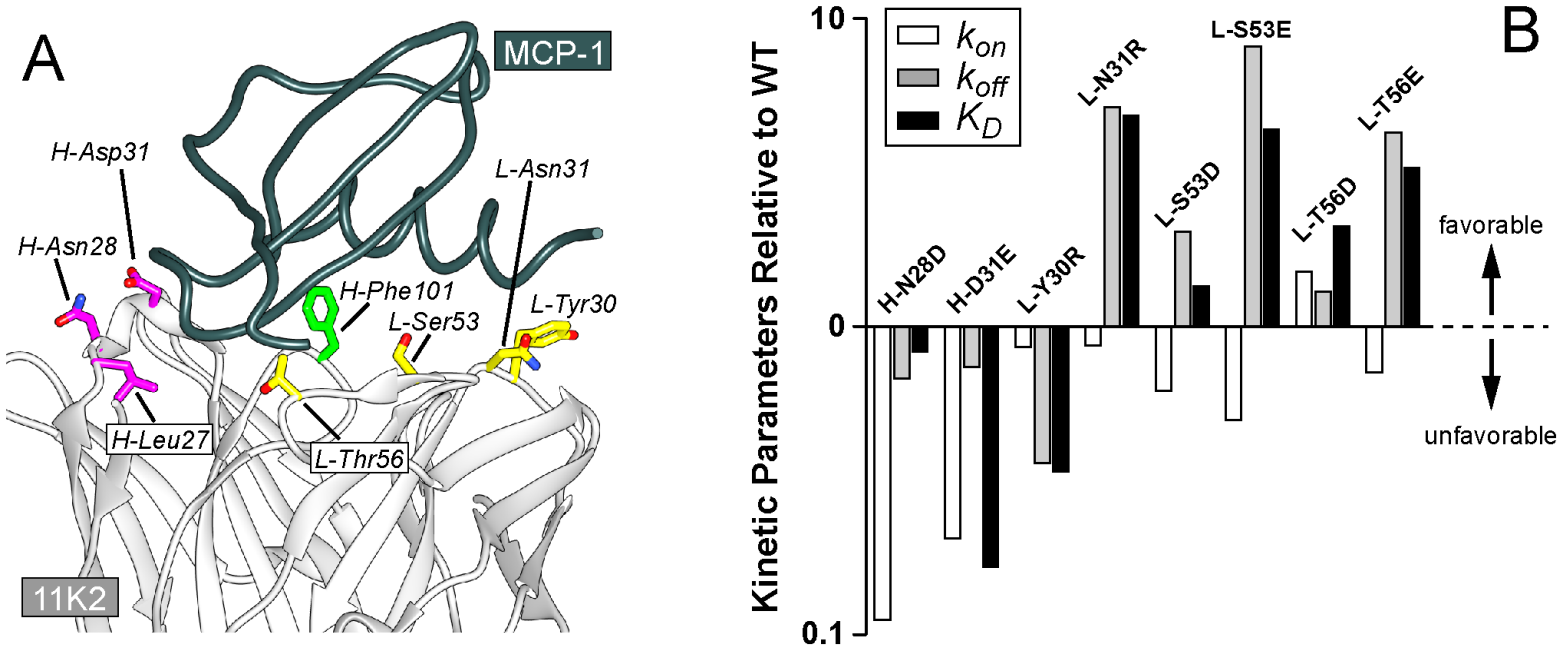
SPR analysis of selected mutations. (**A**) Location of the residues selected in the virtual screening within the crystal structure of the antibody-antigen complex (PDB entry code 2BDN). Mutations belonging to the heavy and light chains are depicted in magenta and yellow, respectively. The antigen is shown in dark green. The hot-spot residue Phe101 is also shown (light green). (**B**) Relative kinetic parameters of the binding of the muteins with respect to wild-type protein. Data is given in Table 1.

The values of *k_on_*, *k_off_*, and *K_D_* were also determined for each mutein (Figure 4, Table 1). Significant differences emerge from the comparison of their binding affinities. Whereas a majority of mutations of the CDR of the V_L_ chain give rise to a robust increase of affinity with respect to the parent antibody (70% of mutations), all mutations belonging to the V_H_ chain destabilize the antibody-antigen complex. Overall, the mutation with the most favorable effect for the affinity is L-N31R. This mutein binds 4.7-fold stronger to the antigen than the wild-type antibody (Figure 3B; *k_on_*  =  13×10^4^ M^−1^ s^−1^; *k_off_*  =  0.22×10^−4^ s^−1^; *K_D_*  =  0.17 nM). Every mutein exhibiting higher affinity for the antigen than that determined for wild-type antibody also displays slower *k_off_* values. In contrast, the destabilizing mutations, without exception, accelerate the dissociation of the antibody from the antigen. Thus the simple examination of *k_off_* predicts the outcome of the mutation in this particular antibody-antigen system.

**Figure 5:**
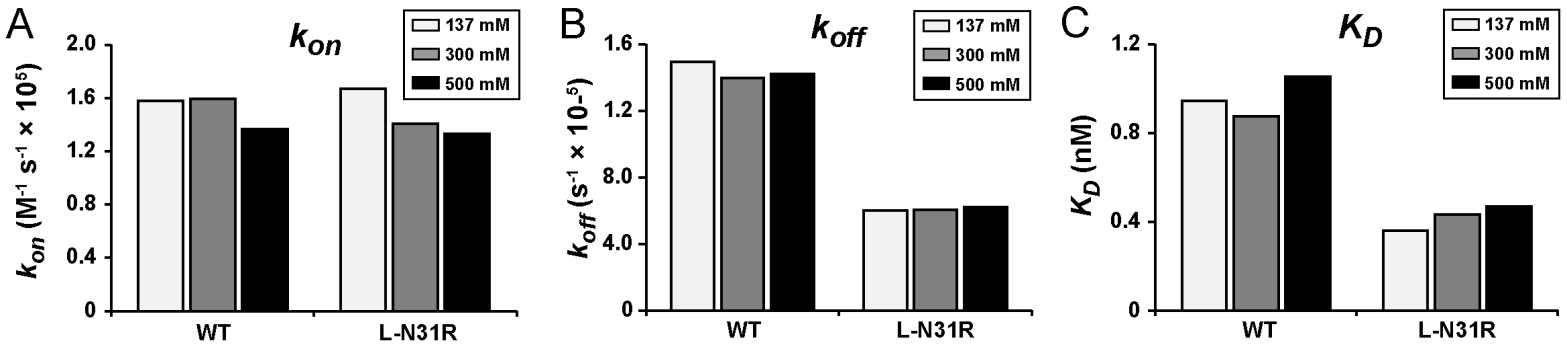
Effect of the ionic strength. (**A**) Association rate constant (*k_on_*), (**B**) dissociation rate constant (*k_off_*), and (**C**) dissociation constant (*K_D_*). The kinetic parameters of the binding of wild-type 11K2 (or mutein L-N31R) to the antigen MCP-1 were determined in running buffer containing three different concentrations of NaCl (137, 300, or 500 mM) at 25°C.

Because the mutein L-N31R (displaying the most favorable effect on affinity) incorporates the positively charged residue Arg we next examined the effect of the ionic strength in three different solutions containing 137, 300 and 500 mM NaCl (Figure 5). No major differences are observed in the kinetic rate constants (*k_on_* or *k_off_*) or the affinity constant (*K_D_*) of wild-type antibody. Similarly, the kinetic parameters do not change dramatically in mutein L-N31R, although we note that the values of *k_on_* decrease slowly but progressively from a value of 17×10^4^ M^−1^ s^−1^ in 137 mM NaCl, to a value of 13×10^4^ M^−1^ s^−1^ in 500 mM NaCl (25% decrease). Similarly, the affinity also decreases by approximately 25% as manifested by an increase of *K_D_* from a value of 0.36 nM to a value of 0.47 nM. The data indicate a modest role of electrostatic interactions, but only during the association (*k_on_*) phase.

### Thermodynamic characterization

**Figure 6:**
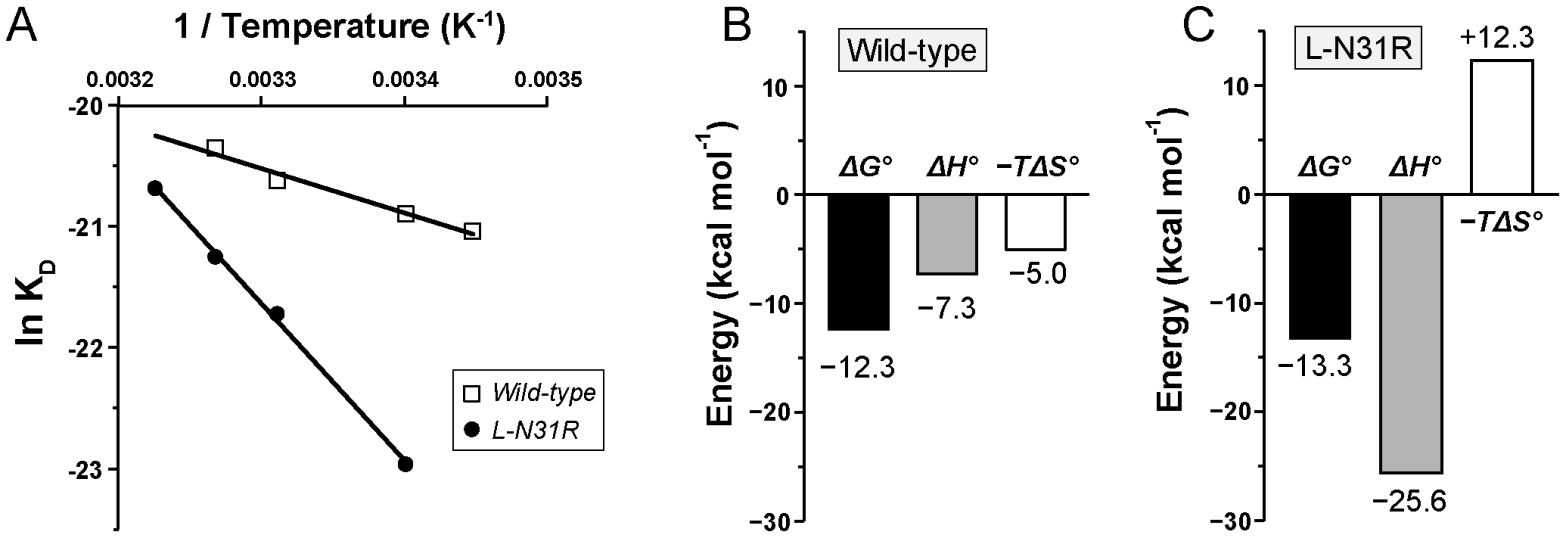
Thermodynamic analysis. (**A**) Regression analysis of the temperature dependence of the dissociation constant *K_D_* yields the van’t Hoff enthalpy (Δ*H°*), entropy (-*T*Δ*S°*) and free energy (Δ*G°*). Empty squares and filled circles correspond to wild-type and L-N31R antibodies, respectively. (**B**) Thermodynamic parameters corresponding to the binding of wild-type antibody to antigen. (**C**) Same parameters obtained for L-N31R.

Thermodynamic parameters for the wild-type antibody and for the optimized muteins were obtained from the temperature dependence of the dissociation constant, as described previously (Figure 6, Table 2) [28], [29]._ENREF_28 The van’t Hoff enthalpy (*ΔH°*) and the entropy (−*TΔS°*, calculated at 25°C) corresponding to the binding of scFv 11K2 to MCP-1 displayed negative values (*ΔH°_WT_*  =  −7.3 kcal mol^−1^, −*TΔS°_WT_*  =  −5.0 kcal mol^−1^) indicating favorable contributions from both energetic terms to the free energy of binding (*ΔG°_WT_*  =  −12.3 kcal mol^−1^). Importantly, the contribution of the enthalpic term increased substantially in the muteins. For example, the value of *ΔH°* of L-N31R is 3.5-fold more favorable to binding than that of wild-type antibody (*ΔH°_L-N31R_*  =  −25.6 kcal mol^−1^, *ΔΔH°_L-N31R_*  =  −18.3 kcal mol^−1^). The change of binding enthalpy of L-N31R is largely (but not completely) compensated by unfavorable changes of entropy (−*TΔS°_L-N31R_*  =  12.3 kcal mol^−1^, −*TΔΔS°_L-N31R_*  =  17.3 kcal mol^−1^, *T*  =  25°C) resulting in a small advantageous change of free energy with respect to the wild-type antibody (*ΔG°_L-N31R_*  =  −13.3 kcal mol^−1^, *ΔΔG°_L-N31R_*  =  −1.0 kcal mol^−1^). Similarly, the other muteins exhibit favorable changes of enthalpy not completely compensated by the entropy term. The thermodynamic analysis clearly demonstrates the favorable contribution of the enthalpy to the improved affinity, suggesting that the mutations generate additional non-covalent interactions between the antigen and antibody in agreement with the *in silico* calculations performed above.

**Table 2:** Thermodynamic parameters of scFv 11K2.

Protein	*ΔH°* (kcal mol^−1^)	*ΔΔH°* (kcal mol^−1^)	–*TΔS°* (kcal mol^−1^)	–*TΔΔS°* (kcal mol^−1^)	*ΔG°* (kcal mol^−1^)	*ΔΔG°* (kcal mol^−1^)
WT	–7.3	0	–5.0	0	–12.3	0
L-N31R	–25.6	–18.3	12.3	17.3	–13.3	–1.0
L- S53D	–13.3	–6.0	0.6	5.6	–12.7	–0.4
L-S53E	–15.1	–7.8	2.4	7.4	–12.7	–0.4
L-T56D	–11.7	–4.4	–1.1	3.9	–12.8	–0.5
L-T56E	–14.5	–7.2	1.9	6.9	–12.6	–0.3

Values of –*T*Δ*S°* and Δ*G°* are given at 25°C.

### Energetic analysis of the transition state

**Figure 7:**
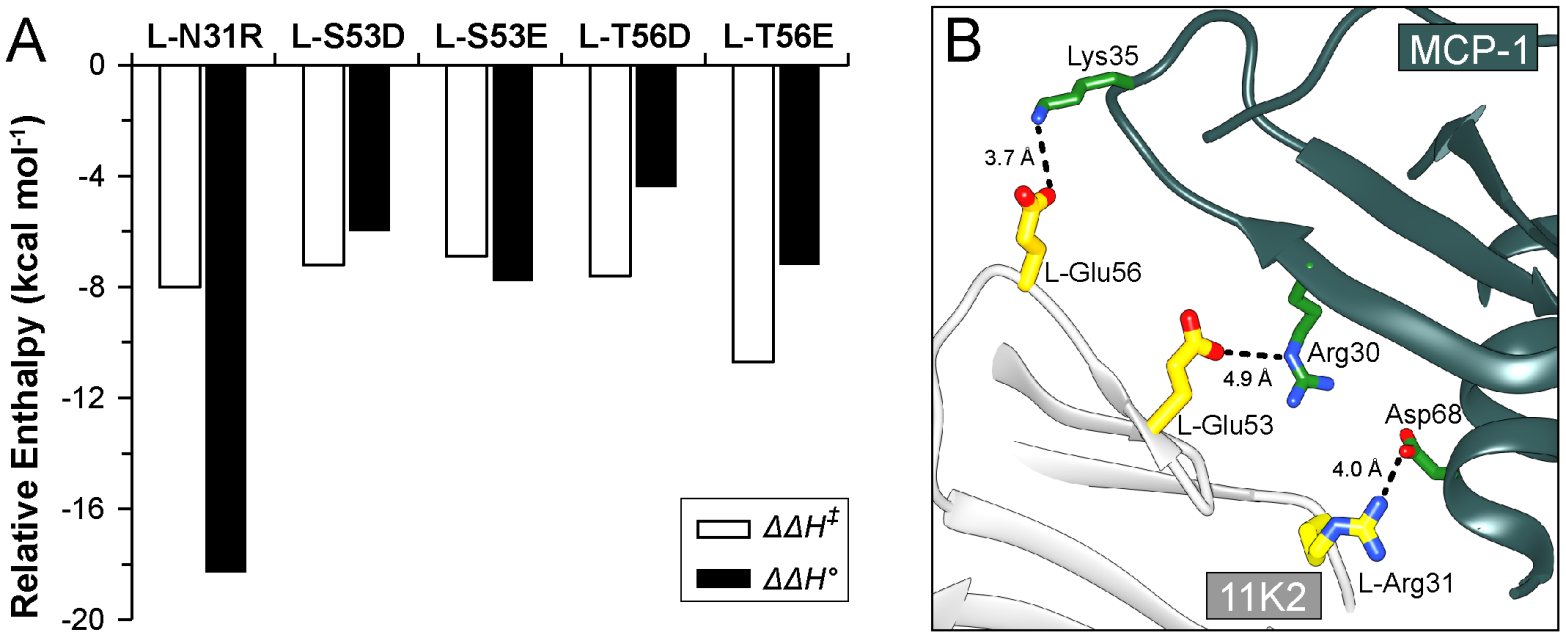
Analysis of the binding enthalpy. (**A**) Favorable changes of binding enthalpy with respect to wild-type antibody at the transition state (empty bar, ΔΔ*H^‡^*) and at equilibrium (filled bar, ΔΔ*H°*). (**B**) Suggested model of the new interactions formed at the antibody/antigen contact surface upon mutation. Residues depicted in yellow and dark green correspond to 11K2 and MCP-1, respectively. The conformation of the side-chain of the mutated residues was modeled from the Dunbrak library of rotamers [44] as implemented in the program Chimera [45] (the most probably rotamer was always selected, except in L-Arg31, where the second most probable rotamer was chosen). Because Lys35 of MCP-1 is not interacting with a neighboring residue in the crystal structure, the conformation of this residue was also modeled as above. The dotted lines and distances represent putative interactions between the mutated residues and the antigen.

The activation energy of each antibody-antigen complex was determined from the temperature dependence of *k_on_* (Table 3, Figure S3). The activation free energy of wild-type antibody is defined by the unfavorable interactions in the transition state (*ΔH^‡^* > 0), reflecting the negative contribution of the dehydration and/or remodeling of protein-protein interactions during the rate determining step (*ΔG^‡^_WT,assoc_*  =  10.6 kcal mol^−1^_,_ *ΔH^‡^_WT,_*_assoc_  =  11.4 kcal mol^−1^, *−TΔS^‡^_WT,assoc_*  =  −0.8 kcal mol^−1^). The relative activation free energy of the muteins does not change significantly with respect to the wild-type antibody (−0.5<*ΔΔG^‡^*_MUT,assoc_<0.3). In contrast, the change of enthalpy of the muteins is more advantageous (less unfavorable) than that of wild-type antibody (−6.9<*ΔΔH^‡^_MUT,_*_assoc_<−10.7 kcal mol^−1^), suggesting the formation of additional non-covalent interactions between the optimized antibody and the antigen during the transition state (Figure 7A). The values of change of enthalpy in the transition state are correlated with the values of change of enthalpy in equilibrium (Figure 7A). The negative values of enthalpy at equilibrium (*ΔΔH°_MUT_*) and at the transition state (*ΔΔH^‡^_MUT,_*_assoc_) demonstrate that the charged residues introduced in the optimized antibody improve the enthalpic contribution to binding. These observations suggest that the charged residues establish electrostatic interactions with the antigen, as depicted in a model of the antibody antigen complex (Figure 7B). The novel interactions are formed early in the complexation reaction, since they play an important role early in the energetic profile of the transition state. In the transition state, these non-covalent interactions are perfectly counterbalanced by unfavorable changes of entropy, reflecting the loss of configurational energy at the rate-limiting step incurred by the approaching proteins (*−TΔΔS^‡^_MUT,assoc_*  =  7.1 ∼ 10.7 kcal mol^−1^, calculated at 25°C) (Figure S4). Although the free energy barrier that the muteins overcome in the transition state is nearly identical within experimental error to that determined for wild-type antibody (*ΔΔG^‡^ ∼ 0*), their energetic pathway towards the antibody-antigen complex in equilibrium differs from each other.

**Table 3:** Activation energy of association of scFv 11K2 to MCP-1.

Protein	*ΔH^‡^* (kcal mol^−1^)	*ΔΔH^‡^* (kcal mol^−1^)	–*TΔS^‡^* (kcal mol^−1^)	–*TΔΔS^‡^* (kcal mol^−1^)	*ΔG^‡^* (kcal mol^−1^)	*ΔΔG^‡^* (kcal mol^−1^)
WT	11.4	0	-0.8	0	10.6	0
L-N31R	3.4	–8.0	6.8	7.6	10.2	–0.4
L-S53D	4.2	–7.2	6.4	7.2	10.6	0
L-S53E	4.5	–6.9	6.4	7.2	10.9	0.3
L-T56D	3.8	–7.6	6.3	7.1	10.1	–0.5
L-T56E	0.7	–10.7	9.9	10.7	10.6	0

Values of –*T*Δ*S^‡^* and Δ*G^‡^* are given at 25°C.

## Discussion

The affinity of the therapeutic antibody 11K2 for its antigen, the inflammatory cytokine MCP-1, was enhanced up to ∼5 fold by employing structure-based computational design. Engineered versions of the antibody were first designed *in silico* and subsequently verified by *in vitro* techniques using a recombinant scFv construct. The detailed thermodynamic characterization revealed the physicochemical principles involved and the operating mechanism.

The selection of suitable candidates of potentially higher affinity among a library of 1,178 virtual mutations was carried out by computational energy minimizations, and the most promising candidates (12 single-mutations) examined experimentally by SPR. Five candidates were mutated at the V_H_ chain, whereas seven candidates were mutated at the V_L_ chain. In five cases the affinity of the optimized antibody increased with respect to the wild-type antibody (42% of muteins tested) − a high success rate. Importantly, only mutations made in the V_L_ chain led to higher affinity (five favorable muteins from a total of seven muteins examined, i.e. 71% success rate). Such domain bias contrasts with other computationally-based optimizations, in which favorable muteins are evenly distributed among V_H_ and V_L_ chains (reviewed in Kuroda *et al*, 2012) [13]. The deleterious effect of mutations at V_H_ for the binding of antigen reflects the excellent optimization of this region in the wild type antibody, both in terms of interaction surface (511 Å^2^ for V_H_; but only 257 Å^2^ for V_L_) and the presence of a hot-spot residue (Figure 4) [18], [30]. In other words, any modification of the V_H_ chain by site-directed mutagenesis disturbs the carefully orchestrated interaction surface with the antigen and consequently reduces the binding affinity.

The single-mutations selected from the virtual screening incorporate, in 92% of the cases, a charged residue. This observation emphasizes the importance of the electrostatic forces in the computational optimization. In two previous studies the optimized antibodies incorporated multiple mutations in their primary sequences [31], [32]. For example, the improvement of the binding affinity of an anti-epidermal growth factor receptor (10-fold) required a triple mutation [31], whereas the optimization of the antibody Y0101 for the antigen VEGF (9-fold) is achieved by incorporating a total of six mutations [32]. Similarly, other examples of antibody engineering not employing electrostatic optimization also required multiple mutations as described in two separate studies (between three and fourteen mutations) [33], [34]. In contrast, our best design achieves a considerable increase of affinity for a matured antibody (5-fold) but requiring only a single-mutation, an approach less likely to alter the three-dimensional structure of the antibody. We have not examined two or more simultaneous mutations of 11K2, an approach that could yield an optimized antibody displaying even higher affinity than that of the single-muteins generated herein.

The enhanced affinity of the optimized 11K2 antibodies is correlated with slower dissociation rate constants (*k*_off_) rather than faster association rate constants (*k*_on_). On the contrary, the destabilizing mutations accelerate the dissociation step, indicating that *k_off_* is a valid parameter to predict the effect of the mutation on the affinity of this particular antibody-antigen complex. A previous report suggested that the dissociation step is a first order reaction whose rate is dictated by the strength of short range interactions between the proteins (van der Waals forces, hydrogen bond, hydrophobic effect, and salt bridges) [35]. Our results convincingly demonstrate that introducing a charged residue in 11K2 increase the affinity for the antigen and slows down *k_off_*.

**Figure 8:**
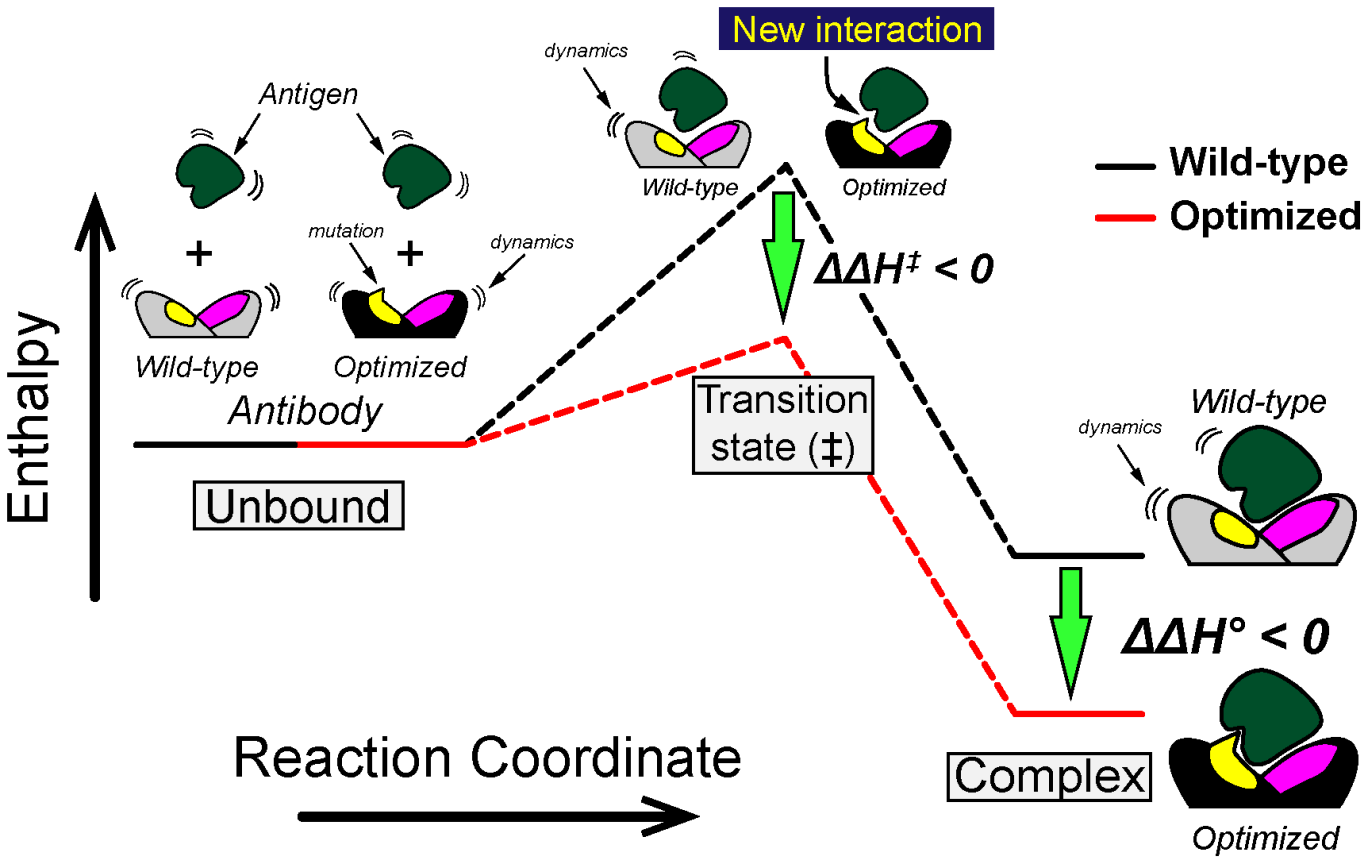
Energetic model of the optimization of an antibody. The diagram represents the enthalpic optimization of an antibody displaying higher affinity for its antigen. The enthalpic advantage acquired during the transition state persists in the complex at equilibrium, although it is largely counterbalanced by the unfavorable contribution of the change of entropy (entropy/enthalpy compensation).

The examination of the thermodynamic parameters (Table 2) indicates that the optimization of the affinity of the antibody-antigen complex is the result of beneficial contributions of enthalpic nature originating in the transition state (*ΔΔH^‡^*<0). It is important to understand the underlying mechanism in terms of the reaction coordinate diagram or “interaction pathway” (Figure 8) [36]–[39]. First, antigen and antibody collide with each other forming the so-called encounter complex (μsec scale) leading to a weakly interacting complex in which the hydration of the protein surface is not altered significantly (not shown) [36], [40]._ENREF_35 The encounter complex progress towards the transition state, although with low efficiency [41]. In the transition state, the antigen-antibody partners lose their hydration layers at the interaction surfaces, and a reconfiguration of intra- and inter-molecular forces takes place. Our results indicate that the beneficial effect of the mutations to the enthalpy in the transition state (*ΔΔH^‡^*<0) is also preserved in the equilibrium complex (*ΔΔH°*<0). In other words, the electrostatic interactions generated in the optimized muteins are actively contributing to binding early in the reaction coordinate, perhaps as early as in the encounter complex, which is a state particularly sensitive to long-range coulombic forces [36]. The new and favorable non-covalent interactions of the optimized muteins are not entirely translated into higher affinity of a similar energetic magnitude, since the entropy/enthalpy compensation effect reduces the influence of the enthalpy advantage in the free energy [42], [43]. Our data indicates that ultimately the enthalpy advantage prevails over the entropic disadvantage in the final antigen-antibody complex albeit with low efficiency since only 5% of the favorable enthalpy is *converted* in useful free energy (Figure S4).

In summary, we illustrate the benefits of using computational methods to design an optimized antibody with higher binding affinity for its cognate antigen. In particular, the incorporation of charged residues improve the affinity by a combination of favorable enthalpic contributions early in the transition state (or even earlier in the encounter complex), and slower dissociation rates (*k_off_*). We hope our study will encourage further investigations into the fundamental physicochemical basis of protein-protein interactions.

## Supporting Information

Figure S1 **Representative examples of energy distribution histograms corresponding to the interaction between 11K2 and MCP-1.** The figure shows the histograms of the wild type (black dotted), the favorable mutein L-N31R (solid, red), and a neutral mutein L-T52S (solid, blue). Muteins displaying favorable histograms with respect to wild-type antibody (i.e. shifted towards the left in the figure above) were selected for additional analysis.
(TIF)

Figure S2 **SDS-PAGE of insoluble fraction of 11K2 scFv.**
(TIF)

Figure S3 **Decomposition of energy terms along the reaction pathway.** The data are given in Table 3 of the manuscript. In each plot, the three energetic levels correspond to the free antibody and antigen, the transition state, and the antibody/antigen complex.
(TIF)

Figure S4 **Entropy/enthalpy compensation plot. (A)** Values at equilibrium. **(B)** Values at the transition state.
(TIF)

Table S1 **DNA and primary sequence of MCP-1 and scFv-11K2.**
(PDF)

## References

[1] Adair JR (1992) Engineering antibodies for therapy. Immunol Rev 130: 5–40. 1286872 10.1111/j.1600-065x.1992.tb01519.x

[2] Lawson AD (2012) Antibody-enabled small-molecule drug discovery. Nat Rev Drug Discov 11: 519–525. 22743979 10.1038/nrd3756

[3] Muda M, Gross AW, Dawson JP, He C, Kurosawa E, et al (2011) Therapeutic assessment of SEED: a new engineered antibody platform designed to generate mono- and bispecific antibodies. Protein Eng Des Sel 24: 447–454. 21498564 10.1093/protein/gzq123

[4] Beck A (2011) Biosimilar, biobetter and next generation therapeutic antibodies. MAbs 3: 107–110. 21285536 10.4161/mabs.3.2.14785 PMC3092613

[5] Kawa S, Onda M, Ho M, Kreitman RJ, Bera TK, et al (2011) The improvement of an anti-CD22 immunotoxin: conversion to single-chain and disulfide stabilized form and affinity maturation by alanine scan. MAbs 3: 479–486. 22048691 10.4161/mabs.3.5.17228 PMC3225852

[6] Constantinou A, Epenetos AA, Hreczuk-Hirst D, Jain S, Deonarain MP (2008) Modulation of antibody pharmacokinetics by chemical polysialylation. Bioconjug Chem 19: 643–650. 18307285 10.1021/bc700319r

[7] Hagihara Y, Saerens D (2012) Improvement of single domain antibody stability by disulfide bond introduction. Methods Mol Biol 911: 399–416. 22886265 10.1007/978-1-61779-968-6_24

[8] Renaut L, Monnet C, Dubreuil O, Zaki O, Crozet F, et al (2012) Affinity maturation of antibodies: optimized methods to generate high-quality ScFv libraries and isolate IgG candidates by high-throughput screening. Methods Mol Biol 907: 451–461. 22907368 10.1007/978-1-61779-974-7_26

[9] Acchione M, Kwon H, Jochheim CM, Atkins WM (2012) Impact of linker and conjugation chemistry on antigen binding, Fc receptor binding and thermal stability of model antibody-drug conjugates. MAbs 4: 362–372. 22531451 10.4161/mabs.19449 PMC3355488

[10] Shiroishi M, Tsumoto K, Tanaka Y, Yokota A, Nakanishi T, et al (2007) Structural consequences of mutations in interfacial Tyr residues of a protein antigen-antibody complex. The case of HyHEL-10-HEL. J Biol Chem 282: 6783–6791. 17166830 10.1074/jbc.M605197200

[11] Barderas R, Desmet J, Timmerman P, Meloen R, Casal JI (2008) Affinity maturation of antibodies assisted by in silico modeling. Proc Natl Acad Sci U S A 105: 9029–9034. 18574150 10.1073/pnas.0801221105 PMC2449359

[12] Park H, Jeon YH (2011) Free energy perturbation approach for the rational engineering of the antibody for human hepatitis B virus. J Mol Graph Model 29: 643–649. 21159534 10.1016/j.jmgm.2010.11.010

[13] Kuroda D, Shirai H, Jacobson MP, Nakamura H (2012) Computer-aided antibody design. Protein Eng Des Sel 25: 507–521. 22661385 10.1093/protein/gzs024 PMC3449398

[14] Kusano KF, Nakamura K, Kusano H, Nishii N, Banba K, et al (2004) Significance of the level of monocyte chemoattractant protein-1 in human atherosclerosis. Circ J 68: 671–676. 15226634 10.1253/circj.68.671

[15] Tominaga T, Miyazaki D, Sasaki S, Mihara S, Komatsu N, et al (2009) Blocking mast cell-mediated type I hypersensitivity in experimental allergic conjunctivitis by monocyte chemoattractant protein-1/CCR2. Invest Ophthalmol Vis Sci 50: 5181–5188. 19553621 10.1167/iovs.09-3637

[16] Hayashida K, Nanki T, Girschick H, Yavuz S, Ochi T, et al (2001) Synovial stromal cells from rheumatoid arthritis patients attract monocytes by producing MCP-1 and IL-8. Arthritis Res 3: 118–126. 11178119 10.1186/ar149 PMC17828

[17] Berman HM, Westbrook J, Feng Z, Gilliland G, Bhat TN, et al (2000) The Protein Data Bank. Nucleic Acids Res 28: 235–242. 10592235 10.1093/nar/28.1.235 PMC102472

[18] Reid C, Rushe M, Jarpe M, van Vlijmen H, Dolinski B, et al (2006) Structure activity relationships of monocyte chemoattractant proteins in complex with a blocking antibody. Protein Eng Des Sel 19: 317–324. 16682434 10.1093/protein/gzl015

[19] Krissinel E, Henrick K (2007) Inference of macromolecular assemblies from crystalline state. J Mol Biol 372: 774–797. 17681537 10.1016/j.jmb.2007.05.022

[20] Kabat E, Wu TT, Perry H, Gottesman K, Foeller C (1991) Sequences of proteins of immunological interest. Bethesda, National Institutes of Health, 2719 p.

[21] Chothia C, Lesk AM (1987) Canonical structures for the hypervariable regions of immunoglobulins. J Mol Biol 196: 901–917. 3681981 10.1016/0022-2836(87)90412-8

[22] Eswar N, Webb B, Marti-Renom MA, Madhusudhan MS, Eramian D, et al.. (2006) Comparative protein structure modeling using Modeller. Curr Protoc Bioinformatics Chapter 5: Unit 5.6. 10.1002/0471250953.bi0506s15 PMC4186674 18428767

[23] Nagao C, Izako N, Soga S, Khan SH, Kawabata S, et al (2012) Computational design, construction, and characterization of a set of specificity determining residues in protein-protein interactions. Proteins 80: 2426–2436. 22674858 10.1002/prot.24127

[24] Umetsu M, Tsumoto K, Hara M, Ashish K, Goda S, et al (2003) How additives influence the refolding of immunoglobulin-folded proteins in a stepwise dialysis system. Spectroscopic evidence for highly efficient refolding of a single-chain Fv fragment. J Biol Chem 278: 8979–8987. 12519771 10.1074/jbc.M212247200

[25] Tsumoto K, Shinoki K, Kondo H, Uchikawa M, Juji T, et al (1998) Highly efficient recovery of functional single-chain Fv fragments from inclusion bodies overexpressed in Escherichia coli by controlled introduction of oxidizing reagent—application to a human single-chain Fv fragment. J Immunol Methods 219: 119–129. 9831393 10.1016/s0022-1759(98)00127-6

[26] Morton TA, Myszka DG (1998) Kinetic analysis of macromolecular interactions using surface plasmon resonance biosensors. Methods Enzymol 295: 268–294. 9750223 10.1016/s0076-6879(98)95044-3

[27] Ross PD, Subramanian S (1981) Thermodynamics of protein association reactions: forces contributing to stability. Biochemistry 20: 3096–3102. 7248271 10.1021/bi00514a017

[28] Sakamoto S, Caaveiro JM, Sano E, Tanaka Y, Kudou M, et al (2009) Contributions of interfacial residues of human Interleukin15 to the specificity and affinity for its private alpha-receptor. J Mol Biol 389: 880–894. 19406127 10.1016/j.jmb.2009.04.050

[29] Cleland WW, Northrop DB (1999) Energetics of substrate binding, catalysis, and product release. Methods Enzymol 308: 3–27. 10506998 10.1016/s0076-6879(99)08003-9

[30] Lutgens E, Faber B, Schapira K, Evelo CT, van Haaften R, et al (2005) Gene profiling in atherosclerosis reveals a key role for small inducible cytokines: validation using a novel monocyte chemoattractant protein monoclonal antibody. Circulation 111: 3443–3452. 15967845 10.1161/CIRCULATIONAHA.104.510073

[31] Lippow SM, Wittrup KD, Tidor B (2007) Computational design of antibody-affinity improvement beyond in vivo maturation. Nat Biotechnol 25: 1171–1176. 17891135 10.1038/nbt1336 PMC2803018

[32] Marvin JS, Lowman HB (2003) Redesigning an antibody fragment for faster association with its antigen. Biochemistry 42: 7077–7083. 12795603 10.1021/bi026947q

[33] Clark LA, Boriack-Sjodin PA, Eldredge J, Fitch C, Friedman B, et al (2006) Affinity enhancement of an in vivo matured therapeutic antibody using structure-based computational design. Protein Sci 15: 949–960. 16597831 10.1110/ps.052030506 PMC2242497

[34] Midelfort KS, Hernandez HH, Lippow SM, Tidor B, Drennan CL, et al (2004) Substantial energetic improvement with minimal structural perturbation in a high affinity mutant antibody. J Mol Biol 343: 685–701. 15465055 10.1016/j.jmb.2004.08.019

[35] Selzer T, Albeck S, Schreiber G (2000) Rational design of faster associating and tighter binding protein complexes. Nat Struct Biol 7: 537–541. 10876236 10.1038/76744

[36] Schreiber G, Haran G, Zhou HX (2009) Fundamental aspects of protein-protein association kinetics. Chem Rev 109: 839–860. 19196002 10.1021/cr800373w PMC2880639

[37] Northrup SH, Boles JO, Reynolds JC (1988) Brownian dynamics of cytochrome c and cytochrome c peroxidase association. Science 241: 67–70. 2838904 10.1126/science.2838904

[38] Schreiber G (2002) Kinetic studies of protein-protein interactions. Curr Opin Struct Biol 12: 41–47. 11839488 10.1016/s0959-440x(02)00287-7

[39] Volkov AN, Worrall JA, Holtzmann E, Ubbink M (2006) Solution structure and dynamics of the complex between cytochrome c and cytochrome c peroxidase determined by paramagnetic NMR. Proc Natl Acad Sci U S A 103: 18945–18950. 17146057 10.1073/pnas.0603551103 PMC1748157

[40] James LC, Tawfik DS (2005) Structure and kinetics of a transient antibody binding intermediate reveal a kinetic discrimination mechanism in antigen recognition. Proc Natl Acad Sci U S A 102: 12730–12735. 16129832 10.1073/pnas.0500909102 PMC1200256

[41] Lipschultz CA, Yee A, Mohan S, Li Y, Smith-Gill SJ (2002) Temperature differentially affects encounter and docking thermodynamics of antibody--antigen association. J Mol Recognit 15: 44–52. 11870921 10.1002/jmr.559

[42] Chodera JD, Mobley DL (2013) Entropy-enthalpy compensation: role and ramifications in biomolecular ligand recognition and design. Annu Rev Biophys 42: 121–142. 23654303 10.1146/annurev-biophys-083012-130318 PMC4124006

[43] Baron R, McCammon JA (2013) Molecular recognition and ligand association. Annu Rev Phys Chem 64: 151–175. 23473376 10.1146/annurev-physchem-040412-110047

[44] Dunbrack RL Jr (2002) Rotamer libraries in the 21st century. Curr Opin Struct Biol 12: 431–440. 12163064 10.1016/s0959-440x(02)00344-5

[45] Pettersen EF, Goddard TD, Huang CC, Couch GS, Greenblatt DM, et al (2004) UCSF Chimera—a visualization system for exploratory research and analysis. J Comput Chem 25: 1605–1612. 15264254 10.1002/jcc.20084

