# Supplementary figures and images for: Affinity Improvement of a Therapeutic Antibody by Structure-Based Computational Design: Generation of Electrostatic Interactions in the Transition State Stabilizes the Antibody-Antigen Complex
Source: PLoS One. 2014 Jan 27;9(1):e87099. doi: 10.1371/journal.pone.0087099 (PMC3903617; doi:10.1371/journal.pone.0087099)

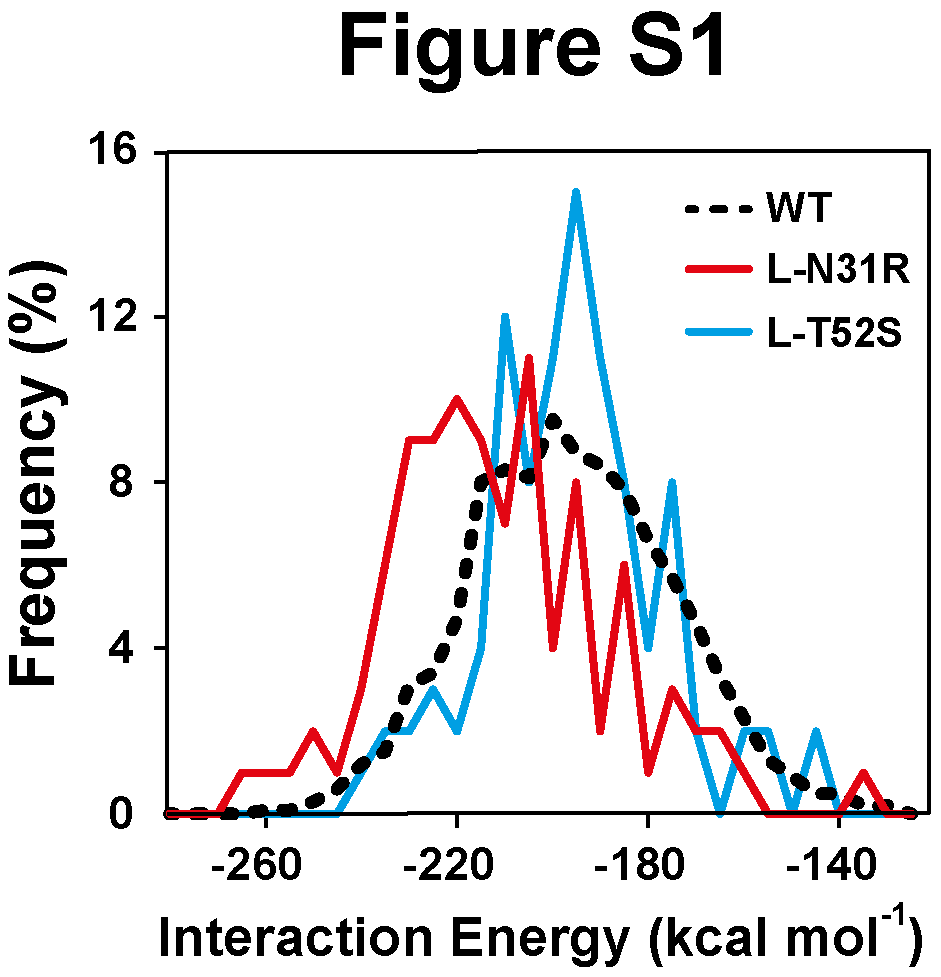

Supplement: Figure S1 — Representative examples of energy distribution histograms corresponding to the interaction between 11K2 and MCP-1. The figure shows the histograms of the wild type (black dotted), the favorable mutein L-N31R (solid, red), and a neutral mutein L-T52S (solid, blue). Muteins displaying favorable histograms with respect to wild-type antibody (i.e. shifted towards the left in the figure above) were selected for additional analysis. (TIF) [file pone.0087099.s001.tif]

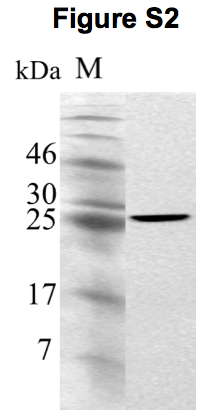

Supplement: Figure S2 — SDS-PAGE of insoluble fraction of 11K2 scFv. (TIF) [file pone.0087099.s002.tif]

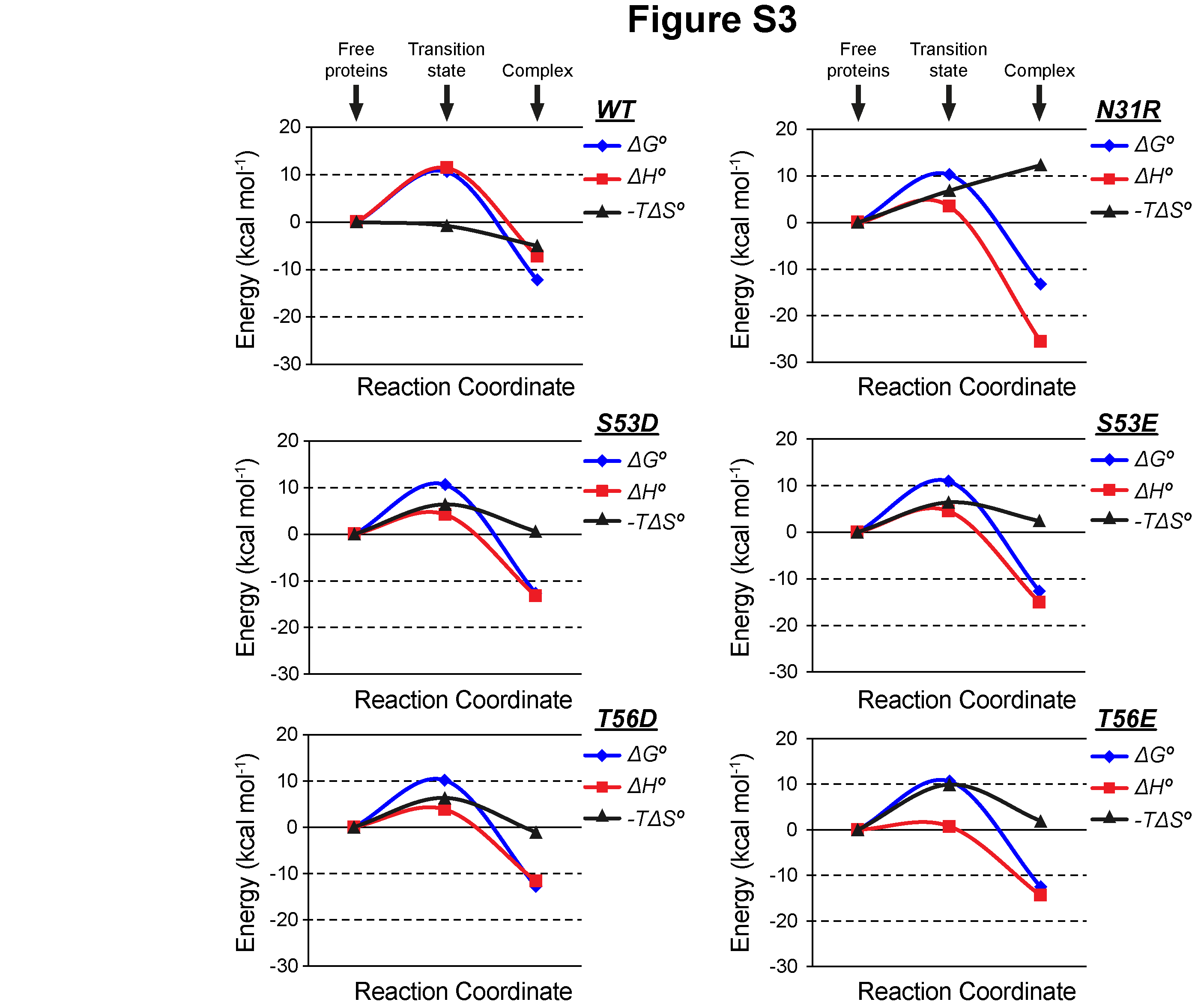

Supplement: Figure S3 — Decomposition of energy terms along the reaction pathway. The data are given in Table 3 of the manuscript. In each plot, the three energetic levels correspond to the free antibody and antigen, the transition state, and the antibody/antigen complex. (TIF) [file pone.0087099.s003.tif]
