# Supplementary material for: Affinity Improvement of a Therapeutic Antibody by Structure-Based Computational Design: Generation of Electrostatic Interactions in the Transition State Stabilizes the Antibody-Antigen Complex
Source: PLoS One. 2014 Jan 27;9(1):e87099. doi: 10.1371/journal.pone.0087099 (PMC3903617; doi:10.1371/journal.pone.0087099)
Supplement: Table S1 — DNA and primary sequence of MCP-1 and scFv-11K2. (PDF) [file pone.0087099.s005.pdf]

Table 1: DNA and primary sequences of MCP-1 and scFv-11K2.

DNA Sequence of MCP-1

ATGCAGCCGGATGCAATTAATGCACCGGTTACCTGCTGTTATAATTTTACCAATCGCAAAATTAGC
GTTCAGCGTCTGGCAAGCTATCGTCGTATTACCAGCAGCAAATGTCCGAAAGAAGCCGTGATTTT
TAAAACCATTGTGGCCAAAGAAATTTGTGCAGATCCGAAACAGAAATGGGTTCAGGATAGCATG
GATCATCTGGATAAACAGACCCAGACCCCGAAAACCCTCGAGCACCACCACCACCACCACTGA

Primary sequence of MCP-1

MQPDAINAPVTCCYNFTNRKISVQRLASYRRITSSKCPKEAVIFKTIVAKEICADPKQKWVQDSMDHL
DKQTQTPKTLEHHHHHH

DNA sequence of scFv-11K2

ATGGAAGTTCAGCTGCAGCAGAGCGGTGCAGAACTGGTTAAAGCCGGTGCAAGCGTTAAACTGA
GCTGTCCGGCAAGCGGTCTGAACATCAAAGATACCTATATGCATTGGGTTAAACAGCGTCCGGAA
CAGGGTCTGGAATGGATTGGTCGTATTGATCCGGCAAATGGCAATACCAAATTTGATCCGAAATT
TCAGGGTAAAGCAACCATTACCGCAGATACCAGCAGCAATACCGCATATCTGCAGCTGAGCAGCC
TGACCAGCGAAGATACCGCAGTTTACTACTGTGCCCGTGGTGTGTTTGGCTTTTTTGATTATTGGG
GTCAGGGCACCACCCTGACCGTTAGCAGCGCGGCCGGCGGGGGCGGTAGCGGCGGTGGCGGGTC
GGGCGGTGGCGGATCGGATGGGGATATCGATATTCAGATGACCCAGAGCAGCAGCAGCTTTAGC
GTTAGCCTGGGTGATCGTGTTACCATTACCTGTAAAGCCACCGAAGATATTTATAATCGCCTGGCA
TGGTATCAGCAGAAACCGGGTAGCGCACCGCGTCTGCTGATTAGCGGTGCAACCAGCCTGGAAAC
CGGTGTTCCGAGCCGTTTTAGCGGTAGCGGTTCTGGTAAAGATTATACCCTGAGCATTACCAGCCT
GCAGACCGAAGATGTTGCAACCTATTATTGCCAGCAGTTTTGGAGCGCACCGTATACCTTTGGTG
GTGGCACCAAACTGGAAATTAAACGTGCAGCCGCGGGTCACCATCATCACCACCATTAA

Primary sequence of scFv-11K2

MEVQLQQSGAELVKAGASVKLSCPASGLNIKDTYMHWVKQRPEQGLEWIGRIDPANGNTKFDPKFQ
GKATITADTSSNTAYLQLSSLTSEDTAVYYCARGVFGFFDYWGQGTTLTVSSAAGGGGSGGGGSGGG
GSDGDIDIQMTQSSSSFSVSLGDRVTITCKATEDIYNRLAWYQQKPGSAPRLLISGATSLETGVPSRFSG
SGSGKDYTLSITSLQTEDVATYYCQQFWSAPYTFGGGTKLEIKRAAAGHHHHHH
